# Investigating incidence of RAS/RAF and PIK3CA alterations in HER2-amplified colorectal cancer: a comprehensive analysis

**DOI:** 10.1093/oncolo/oyaf158

**Published:** 2025-07-31

**Authors:** Svea Cheng, Cyndi Gonzales Gomez, Morgan Ferrell, Richard Giza, Masood Pasha Syed, Tara Magge, Vikram Gorantla, Ronan W Hsieh, Riyue Bao, Aatur Singhi, Anwaar Saeed, Ibrahim Halil Sahin

**Affiliations:** University of Pittsburgh School of Medicine, Division of Hematology/Oncology, Pittsburgh, PA 15213, United States; Department of Internal Medicine, University of Pittsburgh Medical Center, Pittsburgh, PA 15213, United States; Department of Internal Medicine, University of Pittsburgh Medical Center, Pittsburgh, PA 15213, United States; Department of Internal Medicine, University of Pittsburgh Medical Center, Pittsburgh, PA 15213, United States; Department of Internal Medicine, University of Pittsburgh Medical Center, Pittsburgh, PA 15213, United States; UPMC Hillman Cancer Center, Division of Hematology/Oncology at UPMC Shadyside, Pittsburgh, PA 15232, United States; UPMC Hillman Cancer Center, Division of Hematology/Oncology at UPMC Shadyside, Pittsburgh, PA 15232, United States; Division of Hematology and Oncology, University of Washington School of Medicine, Seattle, WA 98109, United States; Department of Internal Medicine, University of Pittsburgh Medical Center, Pittsburgh, PA 15213, United States; UPMC Hillman Cancer Center, Division of Hematology/Oncology at UPMC Shadyside, Pittsburgh, PA 15232, United States; Department of Pathology, University of Pittsburgh School of Medicine, Pittsburgh, PA 15213, United States; Division of Hematology & Oncology, Department of Medicine, University of Pittsburgh Medical Center, Pittsburgh, PA 15213, United States; Division of Hematology & Oncology, Department of Medicine, University of Pittsburgh Medical Center, Pittsburgh, PA 15213, United States

**Keywords:** colorectal cancer, human epidermal growth factor receptor-2, HER2 amplification, KRAS, BRAF, PIK3CA, HER2-directed therapy, resistance mechanisms

## Abstract

**Background:**

Amplification of human epidermal growth factor receptor-2 (HER2) can be targeted with HER2-directed combination therapies for patients with colorectal cancer (CRC). Evolving data from clinical trials suggest mutations in KRAS and PIK3CA, downstream effectors of HER2, may confer resistance to HER2 blockade. However, the true incidence of these alterations in HER2-amplified CRC is largely unknown. In this large cohort study, we investigated the incidence of RAS/RAF and PIK3CA alterations among patients with HER2-amplified CRC.

**Methods:**

Twenty-one studies containing CRC specimens as of April 2023 were sampled using cBioPortal for Cancer Genomics. Clinical, specimen, copy number alteration, and somatic mutation data were aggregated and processed to generate ~30 analysis-ready fields encompassing demographic variables, HER2 amplification, and KRAS/NRAS/PIK3CA/BRAF/MAPK1/MAPK3/HER2 mutations.

**Results:**

Among 4823 patients with CRC, the incidence of HER2 amplification was 2.6% (87/4823), with a higher incidence in male, Asian, and Black patients. Among patients with HER2-amplified CRC, the incidence of KRAS, NRAS, and PIK3CA mutations was 21.8% (19/87) (27.9% [17/61] in colon cancer, 7.7% [2/26] in rectal cancer), 3.4% (3/87)(3.3% [2/61] in colon cancer, 3.8% [1/26] in rectal cancer), and 11.5% (10/87) (13.1% [8/61] in colon cancer, 7.7% [2/26] in rectal cancer), respectively. No BRAF, MAPK1, or MAPK3 mutations were identified. Notably, concurrent HER2 mutation and amplification occurred at an incidence of 16.1% (14/87) (16.4% [10/61] in colon cancer, 15.4% [4/26] in rectal cancer). Median overall survival for all stage patients was significantly lower in patients with HER2-amplified CRC (37.2 months) than in patients with CRC without HER2 amplification (74.9 months) (*P* = .038).

**Conclusions:**

RAS, PIK3CA, and HER2 mutations can commonly co-occur with HER2 amplification, with higher rates in colon cancer than rectal cancer. These findings underscore biological heterogeneity and the importance of molecular profiling in identifying potential resistance before initiation of HER2-directed therapy.

**Implications for Practice**

Our study provides clinical and molecular information on the frequency of potential resistance mechanisms and their relationship with the clinical characteristics of disease. Our study highlights the importance of comprehensive molecular profiling to assess potential biomarkers of resistance before initiation of HER2-directed systemic therapy.

## Introduction

In the United States, colorectal cancer (CRC) is the third most common cancer in both men and women and the second most common cause of cancer-related death overall.^1^ Recent advances in genomic profiling techniques have bolstered our understanding of the molecular underpinnings of CRC, allowing for more targeted and individualized therapies. Such genomic techniques have helped identify multiple potentially actionable genomic alterations in CRC, including human epidermal growth factor receptor-2 (HER2) amplification.^2^

HER2, a member of the epidermal growth factor receptor family, is a transmembrane glycoprotein with tyrosine kinase activity. Its activity is dependent on heterodimerization with other epidermal growth factor receptor members, such as HER3 and EGFR, as it lacks its own ligand and functions as an amplifier of other EGFR signaling. When activated with other receptors, it initiates multiple signaling pathways leading to epithelial cell growth, proliferation, and survival.^3^ Overexpression of HER2 results in increased receptor tyrosine kinase activity and upregulation of downstream pathways, including the mitogen-activated protein kinase (MAPK) and phosphoinositide 3-kinase (PI3K) pathways.^3,4^ Through these mechanisms, HER2 amplification drives tumor development and progression. Not surprisingly, genomic amplification of HER2 has been described in many cancers, including breast cancer, gastric/gastroesophageal cancers, ovarian cancer, endometrial cancer, and CRC. Although the exact prevalence of HER2 amplification in CRC is not well known, some studies report that 3%–4% of metastatic CRC cases are HER2-amplified,^5,6^ leading to worse prognosis and outcomes.^7^

In the last few decades, HER2-directed therapies, such as trastuzumab, a humanized monoclonal antibody targeting the HER2 receptor, have dramatically transformed outcomes for patients with HER2-positive breast and stomach cancers.^8^ By comparison, HER2-targeted therapies in CRC have been less of a focus until recent years. Recent clinical trials have demonstrated that HER2 amplification in metastatic CRC is highly actionable with targeted therapies such as monoclonal antibodies, tyrosine kinase inhibitors (TKIs), and antibody–drug conjugates.^9^ Specifically, these trials found that trastuzumab in combination with HER2-specific TKIs (lapatinib or tucatinib) or another monoclonal antibody (Pertuzumab) was a promising approach to HER2-amplified CRC.^9,10^ However, further clinical trials have shown that oncogenic alterations in pathways downstream of HER2 may render resistance to HER2 blockade.^11^ For example, HER2-amplified CRC with gain-of-function mutations in the RAS and PIK3CA oncogenes may evade molecular inhibition of receptors and, therefore, may not be dependent on HER2 stimulation.

However, the frequency of RAS/RAF and PIK3CA alterations in HER2-amplified CRC is largely unknown. Therefore, in this study, we characterized the clinical and molecular features of HER2 amplification in CRC and investigated the differential incidence of RAS/RAF and PIK3CA alterations in patients with HER2-amplified colon and rectal cancer.

## Methods

The cBioPortal for Cancer Genomics was utilized to obtain data and graphics on HER2 amplification in CRC. A total of 21 studies from cBioPortal were queried for this study (Table S1). Data on patient characteristics, copy number alterations, and somatic mutations were obtained, yielding 77 822 attributes (rows). This dataset was de-duplicated by keeping 1 unique attribute with the most complete data per patient, yielding 48 906 attributes with 1 attribute per patient. We then selected the 28 936 patients with ERBB2 (alias HER2) copy number variation data available. Data were further filtered based on “Location” (rectal or colon), selecting for patients with “Colon Cancer,” “Colorectal Cancer,” or “Colorectal Carcinoma,” resulting in a dataset of 7179 patients for subsequent analysis.

Clinical variables of interest were extracted from the study cohort, including patient age, gender, race, ethnicity, cancer stage, and cancer location. Survival outcome data were extracted, with 0 representing “censored,” “disease free,” “not progressed,” or “living” datapoints and 1 representing “progressed,” “progression,” or “deceased” datapoints. ERBB2 amplification status was extracted from copy number alteration tables, where 5 discrete putative copy number alteration levels were used: “−2” indicates a deep loss, possibly a homozygous deletion; “−1” is a single-copy loss (heterozygous deletion); “0” is diploid; “1” indicates a low-level gain; and “2” is a high-level amplification. For this study, we used “2” (high-level amplification) to select for tumors with ERBB2 amplification. More detailed methodology can be found in the Supplementary Materials section.

## Results

Among a cohort of patients with CRC, the median age was 63 (interquartile range [IQR]: 52–72), 44.4% (2140/4823) were female, and 55.6% (2683/4823) were male. Racially, 11.2% (298/2651) were Black, 8.6% (228/2651) were Asian, 79.9% (2118/2651) were White, and 0.3% (7/2651) were other. In terms of location of CRC, 69.9% (2360/3375) had cancer of the colon and 30.1% (1015/3375) had cancer of the rectum (Table 1).

**Table 1.** Demographics of overall cohort of patients with CRC compared to subset of patients with HER2-amplified CRC.

Characteristic		Patients with CRC	Patients with HER2-amplified CRC
Age: median [IQR]		63 [52–72]	63 [50–77]
Gender	Male	55.6% (2683/4823)	66.7% (82/123)
	Female	44.4% (2140/4823)	33.3% (41/123)
Race	Black	11.2% (298/2651)	12.0% (9/75)
	Asian	8.6% (228/2651)	12.0% (9/75)
	White	79.9% (2118/2651)	76.0% (57/75)
	Other	0.3% (7/2651)	0.0% (0/75)
Cancer location	Colon	69.9% (2360/3375)	70.1% (61/87)
	Rectum	30.1% (1015/3375)	29.9% (26/87)

Abbreviations: CRC, colorectal cancer; IQR, interquartile range; HER2, human epidermal growth factor receptor-2.

**Figure 1.**
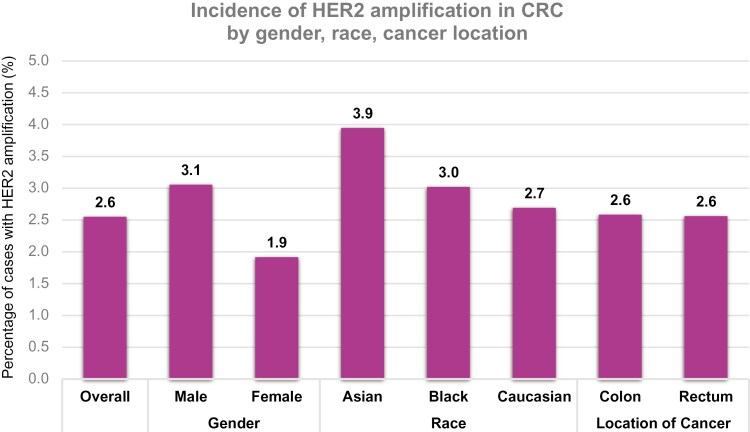
Incidence of HER2 amplification by gender, race, and gender location. HER2, human epidermal growth factor receptor-2.

The incidence of HER2 amplification within this cohort of patients with CRC was 2.6% (123/4823). Among patients with HER2-amplified CRC, the median age was 63 (IQR: 50–77), and 66.7% (82/123) were male. In this cohort, 12% (9/75) of patients were Black, 12% (9/75) were Asian, and 76% (57/75) were White. Notably, 70.1% (61/87) of patients with HER2-amplified CRC had cancer of the colon, and 29.9% (26/87) had cancer of the rectum (Table 1). HER2 amplification incidence was noted to be higher in males (3.1%, 82/2683) than in females (1.9%, 41/2140). When examining HER2 amplification by race, we found that the incidence of HER2 amplification was highest in Asian patients with CRC (3.9%, 9/228), followed by Black patients with CRC (3.0%, 9/298), and then Caucasian patients with CRC (2.7%, 57/2118). Rates of HER2 amplification were similar in the colon and the rectum at 2.6% (61/2360 and 26/1015, respectively) (Table 2, Figure 1).

**Table 2.** Incidence of HER2 amplification by gender, race, and gender location

Characteristic		HER2-amplified cases	Total cases	Incidence of HER2 amplification (%)
Overall		123	4823	2.6
Gender	Male	82	2683	3.1
	Female	41	2140	1.9
Race	Asian	9	228	3.9
	Black	9	298	3.0
	Caucasian	57	2118	2.7
Cancer location	Colon	61	2360	2.6
	Rectum	26	1015	2.6

Abbreviation: HER2, human epidermal growth factor receptor-2.

**Figure 2.**
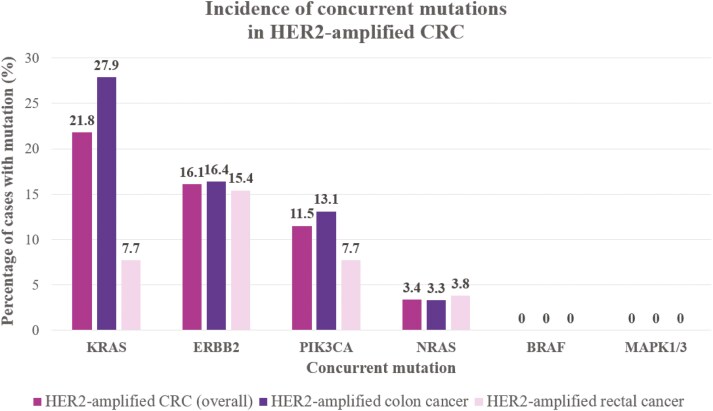
Incidence of concurrent RAS/RAF, PIK3CA, MAPK, and ERBB2 mutations in HER2-amplified CRC. CRC, colorectal cancer; HER2, human epidermal growth factor receptor-2.

After analyzing rates of concurrent MAPK and mTOR pathway mutations in patients with HER2-amplified CRC, it was found that the rates of these mutations varied based on the location of the cancer (Figure 2). KRAS mutations were noted to be the most common mutation in HER2-amplified CRC (21.8%, 19/87), with rates notably higher in HER2-amplified colon cancer (27.9%, 17/61) and lower in HER2-amplified rectal cancer (7.7%, 2/26). PIK3CA mutations were also common, with an overall incidence of 11.5% (10/87) and incidences of 13.1% (8/61) and 7.7% (2/26) in HER2-amplified cancers of the colon and rectum, respectively. NRAS mutations had an overall incidence of 3.4% (3/87) in HER2-amplified CRC, occurring at comparable, low rates in HER2-amplified colon cancer (3.3%, 2/61) and rectal cancer (3.8%, 1/26). No BRAF mutations were reported in either disease group, indicating a distinct molecular feature of HER2-amplified CRC compared to microsatellite stable CRC without HER2 amplification. Similarly, no concurrent MAPK1 or MAPK3 mutations were reported among patients with HER2 amplification. We also examined the rates of co-occurrence HER2 mutations in this population and identified that the overall incidence of concurrent ERBB2 mutation in patients with HER2-amplified CRC was 16.1% (14/87). Rates of concurrent ERBB2 mutation were slightly higher in HER2-amplified colon cancer (16.4%, 10/61) than in HER2-amplified rectal cancer (15.4%, 4/26).

Survival analysis revealed a median overall survival (mOS) of 37.2 months from diagnosis for patients with HER2-amplified CRC (*n* = 75), which was significantly lower than the mOS of 74.9 months for patients with CRC without HER2 amplification (*P* = 0.038).

## Discussion

HER2-amplified colorectal cancer represents a unique molecular subgroup, and it is now among actionable molecular alterations with several precision medicine agents. In this study, we investigated the clinical and molecular characteristics of HER2 amplification with a focus on MAPK pathway alterations. In our study, HER2 amplification occurred at comparable rates in cancers of the colon and rectum. Notably, we identified that the incidence of HER2 amplification varied by gender, with a higher incidence in males than females with CRC. We also found higher incidences of HER2 amplification in Asian and Black patients with CRC compared to their Caucasian counterparts. Moreover, we identified that KRAS/NRAS and PIK3CA alterations are relatively common among patients with HER2-amplified CRC. These somatic events appear to be more common in HER2-amplified colon cancer than rectal cancer. Finally, we identified that 16% of patients with HER2-amplified CRC concurrently had HER2 mutations.

A recent report by Lee et al. also assessed the incidence of RAS/RAF alterations among patients with HER2-amplified CRC as a single institutional experience by using pathology reports from a South Korean hospital.^12^ This report noted a HER2 amplification incidence of 4.1%, notably higher than the 2.6% HER2 amplification incidence in our present study, likely due to demographic differences. This discrepancy in HER2 amplification incidence is consistent with the demographic variation noted in our study, with Asian patients with CRC demonstrating higher HER2 amplification rates (3.9%) compared to Black and White patients with CRC (3.0% and 2.7%, respectively). The Lee et al. report also demonstrated similar rates of KRAS mutations (24.4%) in HER2-amplified CRC compared to our study (21.8%) and no BRAF mutations, similar to our study.^12^ While their report did not note any NRAS mutations, we noted an NRAS incidence of 3.4% in HER2-amplified CRC, likely detected due to the fact that our study represents a larger and multi-institutional patient population.

Moreover, several recent phase 2 trials investigated therapeutic options for patients with HER2-amplified CRC. MyPathway trial was one of the clinical trials investigating biological agents that target HER2 amplification in patients with CRC.^11^ In this study, investigators enrolled patients with mCRC and HER2 amplification regardless of RAS status and investigated the efficacy of trastuzumab and pertuzumab combination. This study revealed an overall objective response rate (ORR) of 32%, although ORR was much higher in patients with wild-type KRAS (40%) compared to patients with a KRAS mutation (8%). Notably, ORR was only 13% among patients with PIK3CA mutation, indicating potential resistance mechanisms. Similar observations were noted in the TRIUMP trial^13^ in which patients with HER2-amplified CRC detected by liquid NGS were treated with trastuzumab and pertuzumab. Investigators showed marked enrichment of RAS/RAF/PIK3CA mutations among non-responders.^13^ The HERACLES trial, which investigated the combination of trastuzumab and lapatinib, did not include patients with codon 12/13 mutations (exon 2 mutations) due to resistance noted in both preclinical and clinical studies.^14^ The Mountaineer trial investigated the combination of trastuzumab and tucatinib and excluded patients with any RAS mutation (including NRAS) and reported an ORR of 38% and median PFS of 8.2 months. Most recently, the DESTINITY CRC-02 investigated trastuzumab-deruxtecan among patients with HER2-amplified CRC.^15^ In this study, patients with KRAS-mutant HER2-amplified CRC were enrolled and noted to have a modest response with 5.4 mg/kg dosing with an ORR of 28%, compared to ~40% among patients with RAS wild-type disease.^16^ Altogether, these studies indicate biomarker selection is key for HER2 targeting in CRC. Our cohort study represents the largest cohort study to date for patients with HER2 amplification, and we identified frequent KRAS/PIK3CA mutations in HER2-amplified CRC. Interestingly, these mutations were more enriched among patients with colon cancer than rectal cancer, representing a distinct biology of colon versus rectal cancer. Although the biomarker value has not been well investigated, we also identified frequent copresence of HER2 mutations among patients with HER2-amplified CRC. Our work further underscores the importance of molecular profiling in HER2-amplified CRC, specifically highlighting how it is critical to not only determine HER2 status but also evaluate for RAS/RAF/PIK3CA mutations using next-generation molecular profiling. Such genetic profiling may help predict the efficacy of different treatments and guide therapeutic decisions and future drug development.

Our survival analysis is consistent with the literature in that HER2-amplified CRC was found to have worse median survival than HER2-non-amplified CRC. However, the data is heterogeneous and may indicate median survival from CRC diagnosis rather than from stage IV CRC diagnosis, which may account for the greater-than-expected median survival. Nonetheless, HER2-amplified CRC often exhibits a more aggressive biology with chemotherapy resistance and intracranial metastasis, leading to treatment failures and resistance.^17^ Chemotherapy resistance has been reported among patients with HER2-amplified CRC, indicating that more advanced research is needed to improve outcomes for this patient population.^18^ Currently, precision therapeutics are being investigated as first-line treatments to deliver targeted therapy early in the disease course (NCT05253651; MOUNTAINEER-03). Additional translational and biomarker research is warranted to enhance the durability of response with HER2-targeting agents. One such approach includes using a combination of precision therapeutics to halt the activation of resistance mechanisms, including MAPK pathway alterations.

Collectively, our study uncovers the clinical and molecular heterogeneity of HER2-amplified CRC and characterizes the relatively frequent molecular alterations that are linked to treatment resistance. Our study has several limitations, including the retrospective nature of the study, lack of detailed clinical data, including specific colonic location, exact stage of disease at the time of diagnosis, and treatment history, potential selection bias of cohorts included in the cBioPortal, and finally, limited clinical and pathological staging information along with survival data. A major strength of our study is that it represents one of the largest cohort studies to date that comprehensively characterizes the clinical and molecular features of HER2-amplified CRC, a relatively rare subgroup of CRC. Further prospective studies are warranted to validate our findings.

## Supplementary Material

oyaf158_suppl_Supplementary_Tables_1

## Data Availability

Data is publicly available in cbioportal, and dataset can be provided upon request.
