# Supplementary material for: Investigating incidence of RAS/RAF and PIK3CA alterations in HER2-amplified colorectal cancer: a comprehensive analysis
Source: Oncologist. 2025 Jul 31;30(7):oyaf158. doi: 10.1093/oncolo/oyaf158 (PMC12311930; doi:10.1093/oncolo/oyaf158)
Supplement: oyaf158_suppl_Supplementary_Tables_1 [file oyaf158_suppl_supplementary_tables_1.docx]

| **Supplemental Table 1: List of 21 studies included in current study cohort** |
| --- |
| • Colon Adenocarcinoma (CaseCCC, PNAS 2015)  • Colon Cancer (CPTAC-2 Prospective, Cell 2019)  • Colon Cancer (Sidra-LUMC AC-ICAM, Nat Med 2023)  • Colorectal Adenocarcinoma (DFCI, Cell Reports 2016)  • Colorectal Adenocarcinoma (Genentech, Nature 2012)  • Colorectal Adenocarcinoma (TCGA, PanCancer Atlas 2018)  • Colorectal Adenocarcinoma Triplets (MSK, Genome Biol 2014)  • Colorectal Cancer (MSK, Cancer Discovery 2022)  • Colorectal Cancer (MSK, Gastroenterology 2020)  • Colorectal Cancer (MSK, JCO Precis Oncol 2022)  • Disparities in metastatic colorectal cancer between Africans and Americans (MSK 2020)  • MSK MetTropism (MSK, Cell 2021)  • Metastatic Colorectal Cancer (MSK, Cancer Cell 2018)  • Rectal Cancer (MSK, Nature Medicine 2022)  • Rectal Cancer (MSK, Nature Medicine 2019)  • MSK-IMPACT Clinical Sequencing Cohort (MSK, Nat Med 2017)  • Cancer Therapy and Clonal Hematopoiesis (MSK, Nat Genet 2020)   - China Pan-cancer (OrigiMed, Nature 2022)   • Pan-cancer analysis of whole genomes (ICGC/TCGA, Nature 2020)  • Ewing Sarcoma (Institut Curie, Cancer Discov 2014)  • Pediatric Ewing Sarcoma (DFCI, Cancer Discov 2014) |
